# High-level gait and balance disorders in the elderly: a midbrain disease?

**DOI:** 10.1007/s00415-013-7174-x

**Published:** 2013-11-08

**Authors:** Adèle Demain, G. W. Max Westby, Sara Fernandez-Vidal, Carine Karachi, Fabrice Bonneville, Manh Cuong Do, Christine Delmaire, Didier Dormont, Eric Bardinet, Yves Agid, Nathalie Chastan, Marie-Laure Welter

**Affiliations:** 1 Centre de Recherche de l’Institut du Cerveau et de la Moelle épiniere (CRICM), Université Pierre et Marie Curie-Paris 6, UMR-S975, Paris, France; 2 Inserm, U975, Paris, France; 3 CNRS, UMR 7225, Paris, France; 4 Groupe Hospitalier Pitié-Salpêtrière, Centre de Neuroimagerie de Recherche (CENIR), Paris, France; 5 Service de Neuroradiologie, Hôpital Rangueil, Toulouse, France; 6 INSERM UMR 825, CHU Purpan, Toulouse, France; 7 UFR STAPS, Université de Paris Sud-11, Orsay, France; 8 Institut des Systèmes Intelligents et de Robotique-ISIR, UPMC, Paris, France; 9 Service de Neuroradiologie, CHRU, Lille, France; 10 Département de Neuroradiologie, Groupe Hospitalier Pitié-Salpêtrière, Assistance Publique-Hôpitaux de Paris, Paris, France; 11 Service de Neurophysiologie, Centre Hospitalier Universitaire de Rouen, Rouen, France; 12 Groupe Hospitalier Pitié-Salpêtrière, Centre d’Investigation Clinique, Assistance Publique-Hôpitaux de Paris, Paris, France; 13 Département de Neurologie, Groupe Hospitalier Pitié-Salpêtrière, Assistance Publique-Hôpitaux de Paris, Paris, France

**Keywords:** Higher-level gait disorders, Gait initiation, MRI, Mesencephalic locomotor region

## Abstract

The pathophysiology of gait and balance disorders in elderly people with ‘higher level gait disorders’ (HLGD) is poorly understood. In this study, we aimed to identify the brain networks involved in this disorder. Standardised clinical scores, biomechanical parameters of gait initiation and brain imaging data, including deep white matter lesions (DWML) and brain voxel-based morphometry analyses, were assessed in 20 HLGD patients in comparison to 20 age-matched controls. In comparison to controls, HLGD patients presented a near-normal preparatory phase of gait initiation, but a severe alteration of both locomotor and postural parameters of first-step execution, which was related to ‘axial’ hypokinetic-rigid signs. HLGD patients showed a significant grey matter reduction in the mesencephalic locomotor region (MLR) and the left primary motor cortex. This midbrain atrophy was related to the severity of clinical and neurophysiologically determined balance deficits. HLGD patients also showed a reduction in speed of gait, related to ‘appendicular’ hypokinetic-rigid signs and frontal-lobe-like cognitive deficits. These last two symptoms were correlated with the severity of DWML, found in 12/20 HLGD patients. In conclusion, these data suggest that the gait and balance deficits in HLGD mainly result from the lesion or dysfunction of the network linking the primary motor cortex and the MLR, brain regions known to be involved in the control of gait and balance, whereas cognitive and ‘appendicular’ hypokinetic-rigid signs mainly result from DWML that could be responsible for a dysfunction of the frontal cortico-basal ganglia loops.

## Introduction

Gait and balance disorders represent a major health problem in the elderly population [1]. The term higher-level gait disorders (HLGD) has been proposed when no specific disease can be identified [2, 3]. HLGD patients show slow gait with shorter strides, poor balance with falls, and gait initiation problems, including freezing of gait [4, 5]. Frontal release and hypokinetic-rigid signs are present, suggesting a dysfunction of the frontal cortex and the basal ganglia [3]. Deep white matter brain lesions (DWML) are observed in about half of cases [5]. In the elderly population, the presence of such lesions has been related to gait disorders and/or falls [6], which has led some authors to propose a vascular origin for this syndrome [1, 7]. However, half of HLGD patients have no DWML [5] and about 20 % of patients with large DWML show neither gait nor balance disorders [1, 8]. These contradictory clinical reports raise the question of the physiological basis of gait and balance disorders in HLGD patients, and suggest the presence of brain lesions or dysfunction in addition to DWML [3, 9].

In mammals, the control of gait and balance involves several brain structures, in particular, the frontal cortico-basal ganglia network, which includes the primary motor cortex, premotor and supplementary motor areas and the basal ganglia. As a key structure, the importance of the midbrain mesencephalic locomotor region (MLR), comprising the pedunculopontine (PPN) and cuneiform nuclei (CN) [9], has recently been highlighted with respect to Parkinson’s disease (PD). Indeed, gait and balance disorders unresponsive to levodopa treatment in parkinsonian patients have been related to midbrain atrophy and loss of PPN cholinergic neurons [10–12], with an improvement of falls with low frequency deep brain stimulation of the PPN area [13]. In healthy volunteers, the MLR has been shown to be activated during both real and imaginary gait and postural control [12, 14–20]. In animals, MLR electrical stimulation modulates muscle tone and elicits treadmill locomotion [21], and specific lesions of the cholinergic PPN neurons in monkeys induce gait and postural deficits [10].

Taking all these data into account, we hypothesised that lesion or dysfunction of the MLR region could induce the gait and balance disorders presented by HLGD patients [4, 5].

In the present study, we used a multidisciplinary approach with clinical assessments, brain imaging, and biomechanical and neurophysiological recordings to better understand the neural substrate of gait and balance disorders in HLGD patients, with the MLR, the basal ganglia and the frontal cortex seen as the most likely candidates.

## Methods

### Subjects

Thirty patients referred to the Neurology Department of the Pitié-Salpêtrière Hospital for levodopa-unresponsive hypokinetic-rigid gait and balance disorders were included in the study (14F/16M, mean age [SD]: 75.5 [9.8] years; mean disease duration [SD]: 4.2 [2.4] years). Inclusion criteria for HLGD were gait and balance disorders with no evidence of rheumatological, orthopaedic or neurological disease that could explain the signs [4, 5]. Exclusion criteria were: motor or sensory lower limb deficit or pyramidal spasticity, cerebellar ataxia, oculomotor palsy, vestibular deficit, pain during walking, resting tremor, parkinsonian symptoms responsive to dopaminergic treatment, orthostatic hypotension, limb apraxia, history of stroke, encephalitis, head injury, loss of consciousness, use of dopamine receptor blocking agents or dementia. Patients with focal brain lesions other than a single lacunar infarct or with possible chronic hydrocephalus were also excluded. All subjects gave informed written consent and the study was approved by the local ethical committee (University Paris VI, INSERM: RBM: 02-60, ClinicalTrials.gov Registration: NCT00139321).

All patients performed the test procedure at the time of inclusion (see below), but after a prospective follow-up of more than 4 years, 20 were diagnosed with HLGD (9F/11M, mean age [SD]: 77.0 [6.8] years; mean disease duration [SD]: 3.9 [2.9] years) and compared to 20 age-matched controls (10F/10M; mean age [SD]: 76.1 [6.6] years). Ten patients were finally excluded because they developed PD (*n* = 2), multiple system atrophy (MSA) (*n* = 3), progressive supranuclear palsy (PSP) (*n* = 1), frontotemporal dementia (*n* = 1), cerebellar ataxia (*n* = 2) or vascular parkinsonism (*n* = 1).

### Test procedure

#### Clinical evaluation

Gait and balance disorders were assessed by both patient interview and objective clinical examination, using the Unified Parkinson Disease Rating Scale (UPDRS III) [22] and the Rating Scale for Gait Evaluation (RSGE) [23]. Individual interview items of particular interest were freezing episodes and falls (items 13 and 14 of the UPDRS, respectively). Objective examination included the assessment of hypokinetic-rigid signs by calculating the ‘axial’ (UPDRS III items: 18-speech + 22a-nuchal rigidity + 28-posture + 29-gait + 30-postural stability, range 0–20) and ‘appendicular’ (UPDRS III total less the ‘axial’, range 0–88) scores [22]. Cerebellar, autonomic, pyramidal and oculomotor functions were also assessed clinically [24]. Cognitive function was examined using the mini-mental status examination (MMSE) and the frontal assessment battery (FAB) [25].

#### Gait initiation walking test

**Fig. 1:**
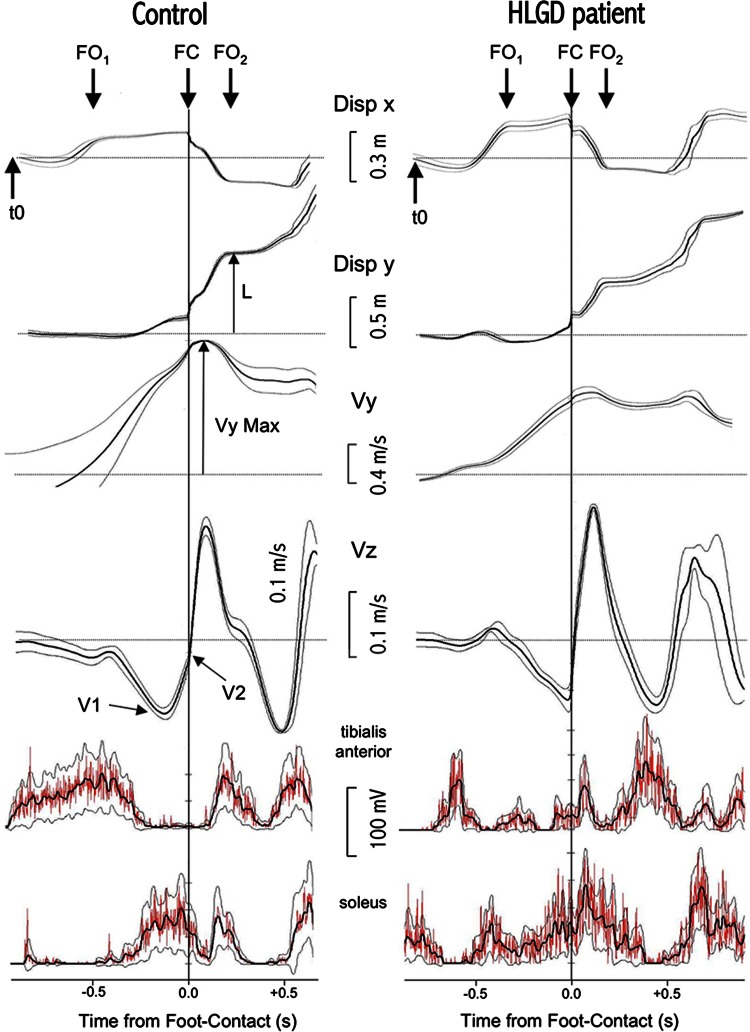
Biomechanical parameters and leg muscle activity during gait initiation in an individual control subject and an HLGD patient in the fast gait condition. From *top* to *bottom*, *curves* represent the smoothed mean of ten trials (Tukey algorithm, ± its 95 % confidence limits) and show the mediolateral (Disp X) and anteroposterior (Disp y) CP displacements, antero-posterior CP velocity (*Vy*) and vertical CG velocity (*Vz*). The smoothed and rectified electromyography (EMG) activity of the anterior tibialis and soleus muscles of the stance leg is shown at the bottom with its 95 % confidence limits (large black trace) superimposed on the unsmoothed mean rectified EMG. The mediolateral (x) displacement of the CP enables the measurement of the lateral displacement of the CP before foot-off (mediolateral APAs) and the step width. The anteroposterior (y) displacement of the CP enables the measurement of the posterior displacement of the CP before the foot-off (anteroposterior APAs), the step length (L), and the speed of the execution of the gait initiation (L/(*t*FC-*t*FO1)). With the anteroposterior velocity of the CG, the maximum forward velocity (*Vy*) was measured at the end of the first step. The CG vertical velocity curve enables us to measure the position of V1 (negative peak of the CG vertical velocity) and V2 (CG vertical velocity at the time of foot-contact) and the braking index ((V1−V2)/V1 × 100). In the control subject shown here, the vertical velocity of the CG describes a V shape indicating a fall in the CG (V1). Just before foot-contact, active braking occurs and the vertical velocity increases (V2). The HLGD patient shows no active braking (V1 = V2). *t0* time of the first biomechanical event, *FO1* foot-off of the swing leg, *FC* foot-contact of the swing leg, *FO2* foot-off of the stance leg

The gait initiation process was specifically studied, as this requires simultaneous forward movement (locomotion) and balance control in order to maintain stability and prevent falling. Locomotion was characterised by the measures of step length (L) and velocity (Vm), and balance control by the measure of both anticipatory postural adjustments (APAs, the period between the first biomechanical event [*t*0] and foot-off of the swing leg [FO1]), that occur before leg lifting, and of the vertical acceleration of the centre of gravity (CG) during the execution of the first step. The vertical velocity of the CG enables us to measure the braking index ([V1−V2]/V1 × 100, where a value below 25 % is considered abnormal) (Fig. 1) [11, 26, 27].

Biomechanical parameters of gait initiation were recorded using a force platform (0.9 × 1.8 m, AMT Inc LG6-4-1). Subjects, barefoot and standing upright, were instructed to commence walking for 5 m following an auditory cue under two conditions: ‘natural’ (usual walking pace) and ‘fast’ (walking as fast as possible taking large steps), with ten trials being performed in each condition. The accelerations and velocities of the CG and centre of foot pressure (CP) displacement of the first two steps were calculated, and the lower limb EMG was concomitantly recorded (Fig. 1) [11].

#### Image acquisition and processing

Among the 40 subjects, 16 HLGD patients were recruited for the magnetic resonance imaging (MRI) study (8F/8M, mean age: 75.7 [5.7] years), because MRI acquisition or processing problems preventing further analysis occurred for four patients. These were compared to 16 controls (8F/7M, mean age: 69.2 [5.9] years). Images were acquired using a standard head coil on a 1.5 T General Electric Signa™ magnet [11]. First, MRI scans were examined for any signal abnormalities (leukoaraiosis or lacunar infarctions) in the white matter. The extent of leukoaraiosis was graded from none (grade 0) to severe (grade 4) [28]. Second, three morphometric analyses were performed: (1) measurement, by manual delineation, of the mesencephalic surface area on T2 images, normalised by division with the brain parenchymal volume (automatically measured on T1 images using the fslstats function, FMRIB’s Software Library) [11]; (2) calculation of the magnetic resonance parkinsonism index (MRPI), as previously described [29], shown to predict the progression of clinically unclassified parkinsonism towards PSP phenotypes [30]; and (3) a voxel-based morphometry (VBM) analysis of T1 images to examine the changes in the grey and white matter concentration with the Statistical Parametric Mapping 8 software package (SPM8, Wellcome Trust Centre for Neuroimaging, London, UK). Anatomical locations of the resulting map at the cortical level were identified with the TalairachClient 2.4.2 [31, 32]. Because of our a priori hypothesis, we also used a small volume correction (SVC), comprising the basal ganglia structures and brain stem (which includes the midbrain, the pons and the superior and middle cerebellar peduncles) by designing two masks. To precisely determine the location of the differences in the VBM analysis, we used a validated three-dimensional (3D) histological and deformable atlas of the basal ganglia and midbrain area that includes the PPN and the CN [13, 20, 33].

#### Statistical analysis

Ten-trial averages were calculated for each gait initiation parameter, and mean values and standard deviations were calculated for each condition.

For biomechanical parameters of gait initiation, comparison of HLGD patients with controls was performed using the non-parametric Mann–Whitney *U*-test. The Wilcoxon Signed-Ranks test was used to compare the natural and fast gait conditions parameters, in both patients and controls. To determine the relationship between clinical, biomechanical and imaging data, we first performed a univariate Spearman correlation test, followed by a stepwise multiple regression analysis for variables found to be significant.

For the imaging data, the SPM8 factorial design specification was used in order to establish the statistical global linear model (*t*-tests) that allows us to compare the two groups. An analysis of covariance (ANCOVA) was designed to investigate focal grey and white matter volume differences between subject groups. Age and intracranial volume were also incorporated as covariates. The level of statistical significance was set as *P* < 0.001 throughout the whole brain, and clusters were ultimately considered significant at *P* < 0.05, corrected (family-wise error-FWE) for multiple comparisons.

## Results

### Clinical features

Twelve out of 20 HLGD patients and 9/20 controls had cardiovascular risk factors (HLGD patients: hypertension: *n* = 8; cardiac infarct: *n* = 2; dyslipidemia: *n* = 4; diabetes: *n* = 1; Controls: hypertension: *n* = 5; dyslipidemia: *n* = 3, diabetes: *n* = 1).

All 20 HLGD patients had hypokinetic-rigid symptoms with mainly ‘axial’ involvement (mean ± SD ‘axial hypokinetic-rigid’ score = 6.5 ± 3.1, RSGE score = 25.5 ± 9.8) and fewer ‘appendicular’ motor signs (mean ± SD ‘appendicular hypokinetic-rigid score’ = 11.1 ± 8.1). Five patients showed a slow decrease in vertical ocular pursuit in the up (*n* = 5) or down direction (*n* = 2) and/or a slow unlimited horizontal pursuit deficit (*n* = 2). Four patients had significant urinary incontinence, one following prostatic surgery. None of the patients displayed pyramidal syndromes, cerebellar ataxia or symptomatic orthostatic hypotension.

### Neuropsychological assessment

The MMSE showed no significant difference between HLGD patients (mean ± SD = 27.6 ± 2.5) and controls (mean ± SD = 28.9 ± 1.2, *P* = 0.06). Conversely, the FAB score was below 15 in 11/20 patients and significantly lower than controls (mean ± SD in HLGD patients = 13.2 ± 3.7 and controls = 17.5 ± 0.6, *P* < 0.01).

### Gait initiation parameters (Table 1)

The mean duration of the APAs and posterior and lateral CP displacements showed no significant difference between HLGD patients and controls. During gait initiation execution, the mean width, length and maximum anteroposterior velocity of the first step were all significantly lower in HLGD patients than controls (Table 1).

**Table 1:** Biomechanical characteristics of gait initiation in 20 HLGD patients and 20 age-matched controls

	Natural gait		*P* value	Fast gait		*P* value
	Control group	HLGD group		Control group	HLGD group	
Anticipatory postural adjustments (*t*0–*t*FO1)						
Lateral CP displacement, mean (SD) (cm)	3.11 (0.67)	3.45 (4.89)	0.23	3.21 (0.56)	3.81 (2.18)	0.001
Posterior CP displacement, mean (SD) (cm)	−4.28 (1.60)	−4.39 (4.56)	0.83	−6.73 (1.63)	−4.94 (2.14)	0.57
APA duration (*t*0–*t*FO1), mean (SD) (ms)	0.60 (0.15)	0.58 (0.14)	0.27	0.59 (0.09)	0.58 (0.18)	0.34
Gait initiation execution (*t*FO1–*t*FC)						
Step width–W, mean (SD) (m)	0.17 (0.04)	0.07 (0.02)	<0.001	0.18 (0.03)	0.08 (0.03)	<0.001
Step length–L, mean (SD) (m)	0.53 (0.08)	0.28 (0.10)	<0.001	0.69 (0.11)	0.42 (0.15)	<0.001
Maximum AP velocity of the CG–Vm, mean (SD) (m/s)	0.87 (0.17)	0.49 (0.17)	<0.001	1.29 (0.22)	0.76 (0.27)	<0.001
CG Fall–V1, mean (SD) (m/s)	−0.11 (0.04)	−0.06 (0.03)	<0.001	−0.21 (0.04)	−0.11 (0.05)	<0.001
CG vertical velocity at foot-contact–V2, mean (SD) (m/s)	−0.06 (0.03)	−0.05 (0.02)	0.07	−0.10 (0.05)	−0.07 (0.05)	0.16
[V1−V2], mean (SD) (m/s)	0.05 (0.03)	0.02 (0.02)	<0.001	0.11 (0.04)	0.03 (0.03)	<0.001
Braking of CG fall, mean (SD) (%)	44.4 (19.5)	28.6 (≈12.9)	0.02	52.8 (19.8)	39.5 (29.4)^a^	0.01
Braking of CG fall duration, mean (SD) (ms)	83.7 (38.2)	98.0 (≈39.8)	0.09	94.0 (20.9)	88.1 (63.8)^a^	0.14
Double stance duration (ΔtFC–*t*FO2) (ms)	231.6 (47.3)	286.1 (70.3)	0.02	176.7 (17.8)	221.7 (65.0)	0.02

^a^ Values were calculated for the four patients with a step length > 35 cm in the natural gait condition and for the 13 patients with a step length > 35 cm in the fast gait condition

*AP* anteroposterior, *APA* anticipatory postural adjustments, *CG* centre of gravity, *FC* foot-contact, *FO* foot-off, *ML* mediolateral

In the natural gait initiation condition, 16/20 patients had step lengths below 35 cm; therefore, a small fall in the CG occurred and no braking was necessary [11]. In the four remaining patients, the braking index was significantly lower than controls. In the fast gait initiation condition, 13/20 patients showed a step length > 35 cm with a persistent decrease in the braking index compared to controls. In patients with impaired braking, leg muscle activation timing was altered, with a delayed activation of the soleus of the stance leg and a concomitant activation of both anterior tibialis and soleus muscles (Fig. 1). The mean gait initiation execution and braking durations ([*t*FC–*t*FO1] and [*t*FC–*t*V1], respectively) showed no significant difference between HLGD patients and controls, but the double-stance duration (*t*FC–*t*FO2) was significantly higher in HLGD patients.

In the fast compared to the natural gait condition, all the gait initiation parameters were higher, except for the duration of APAs, step width, braking index and duration, in both HLGD patients and controls (Table 1).

### Brain lesions and morphometric abnormalities

Single lacunae were found in eight patients (putamen, *n* = 5; pons; *n* = 2, cerebral peduncle, *n* = 1) and four controls (putamen, *n* = 2; pons, *n* = 1; cerebral peduncle, *n* = 1). Lesions in the periventricular white matter were found in 12 HLGD patients (mean grade: 1.3 [1.1]) and six controls (mean grade: 0.6 [0.8], *P* = 0.05). Six patients and three controls also showed small white matter lesions in the pons (mean grade: 0.7 [1.0] and 0.2 [0.4], respectively, *P* = 0.07).

**Fig. 2:**
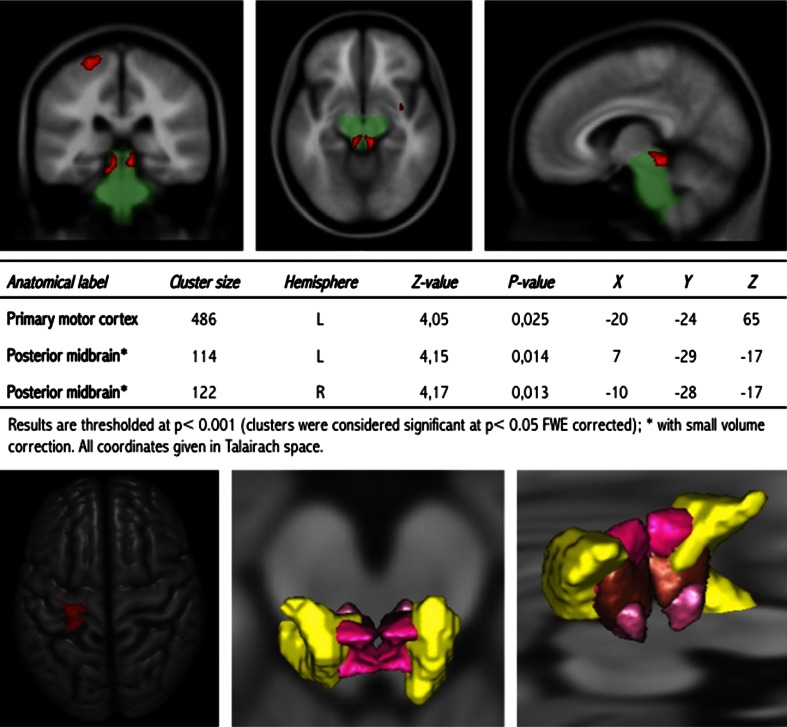
Brain voxel-based morphometry analysis in HLGD patients as compared to controls. *Top* Statistical parametric map of reduced grey matter (*red*) in the left primary motor cortex and MLR superimposed on the MNI152 template. *Left* frontal, *centre* axial and *right* sagittal views. The mask used for the MLR small volume correction analysis is shown in *green*. *Centre* Significant reduction in VBM grey matter analysis in HLGD patients compared to controls. *Bottom* 3D representations of the SPM reduced grey matter. *Left* Anatomical location of the high intensity region of the cortex (*red*). *Centre* and *right*: Anatomical location of the high intensity region of the midbrain (*yellow*) verified using the 3D histological and deformable YeB atlas [20, 33]. This atlas was mapped onto the MNI template through a validated intensity-based deformation procedure adapted for subcortical structures. The structures shown are (from *top* to *bottom*): cuneiform nucleus (*pink*), dorsal PPN (*brown*) and ventral PPN (*purple*)

A significant decrease in grey matter density of the left primary motor cortex and bilaterally in the midbrain was found in HLGD patients using VBM measurement with SVC (Fig. 2). The grey matter decrease was located bilaterally in the posterior region of the midbrain and involved parts of both the CN and the PPN (Fig. 2). No significant difference in grey matter density was found in the pons, superior and middle cerebellar peduncles (Fig. 2), the basal ganglia or the thalamus. No significant difference in white matter density was found between HLGD patients and controls (not shown). The MRP index was 6.4 ± 2.3 in HLGD patients and 5.9 ± 1.4 in healthy controls (*P* = 0.72), with no patient having an MRP index greater than 13.55.

### Relationship between clinical, neurophysiological and imaging data in HLGD patients

The FAB score was significantly correlated to the postural stability and ‘appendicular’ hypokinetic-rigid signs and scores (Table 2) and with the maximum AP-CG velocity (*r* = 0.48, *P* = 0.04, not shown). Age and MMSE scores were not found to be significantly related to any clinical or biomechanical parameters of gait (not shown).

**Table 2:** Correlation coefficients for cognitive status, clinical and biomechanical characteristics of gait and brain imaging data in HLGD patients

	‘Axial’ hypokinetic-rigid signs^a^					‘Appendicular’ hypokinetic-rigid signs^a^	Brain imaging data	
	Freezing of gait	Falls	Axial score	Gait	Postural stability		*N*-mesencephalon surface area	DWML
Cognition (FAB score)	−0.03	−0.36	−0.42	−0.24	**−0.52** ^**b**^	**−0.72** ^**b,c**^	0.03	−0.45
Gait initiation parameters								
Anticipatory postural adjustments								
Lateral CP displacement	**0.48** ^**b**^	**0.49** ^**b**^	**0.67** ^**b, c**^	**0.52** ^**b**^	**0.49** ^**b**^	0.43	**−0.52** ^**b**^	−0.15
Posterior CP displacement	**−0.48** ^**b**^	**−0.52** ^**b**^	**−0.60** ^**b,c**^	**−0.58** ^**b**^	**−0.51** ^**b**^	−0.21	**0.48** ^**b**^	0.15
Gait initiation execution								
Step width	**−0.52** ^**b**^	−0.15	−0.39	**−0.59** ^**b,c**^	−0.40	−0.44	−0.11	−0.11
Step length	−0.06	0.29	−0.37	−0.31	−0.23	−0.16	0.14	0.25
Maximum AP velocity of the CG	−0.03	0.17	−0.42	**−0.48** ^**b**^	−0.34	**−0.52** ^**b**^	−0.08	**−0.49** ^**b**^
Braking of the CG fall	−0.37	0.19	−0.20	−0.02	**−0.48** ^**b,c**^	−0.39	**0.49** ^**b**^	0.33
Double stance duration	−0.20	−0.11	0.26	0.19	0.15	0.15	**−0.50** ^**b**^	−0.17
Brain imaging data								
*N*-mesencephalon surface area	−0.23	**−0.61** ^**b,c**^	0.20	−0.18	**−0.48** ^**b**^	−0.22	–	–
Deep white matter lesions (DWML)	**0.59** ^**b**^	−0.09	0.41	0.43	0.42	**0.73** ^**b,c**^	0.06	–

*FAB* frontal assessment battery, *AP* anteroposterior, *CG* centre of gravity, *CP* centre of foot pressure, *DWML* deep white matter lesions, *N-mesencephalon* normalised-mesencephalon

^a^ See “Patients and methods”.

Entries in bold = ^b^: *P* < 0.05 after univariate analysis (non-parametric Spearman correlation) and ^c^ *P* < 0.05 after multivariate analysis (stepwise multiple regression analysis)

The severity of ‘axial’ hypokinetic-rigid signs, freezing of gait, falls, and postural instability were significantly correlated to the lateral and posterior displacements of the CP during the APAs (Table 2). The freezing of gait score was also significantly related to the step width, the gait score to the step width and the maximum AP-CG velocity, and the postural stability score to the braking index (Table 2). The severity of ‘appendicular’ hypokinetic-rigid signs was only related to the maximum AP-CG velocity (Table 2).

**Fig. 3:**
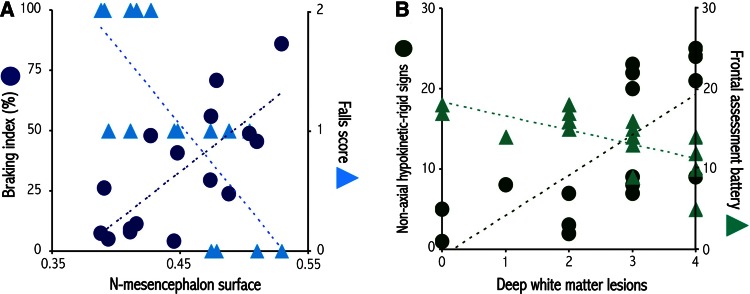
Relationship between biomechanical parameters of gait initiation, clinical gait and balance disorders and brain lesions and atrophy. The *graphs* represent the relationship between **a** the N-mesencephalon surface area and the braking index (*blue circles*) and the falls score (*blue triangles*), **b** deep white matter lesions and ‘appendicular’ hypokinetic-rigid signs (*green circles*) and the frontal assessment battery score (*green triangles*)

The N-mesencephalon surface area was significantly negatively correlated with the falls and postural stability score, the lateral and posterior displacements of the CP during the APAs, the braking index, and double-stance duration (Table 2, Fig. 3a). The severity of DWML was significantly correlated with the severity of freezing of gait and ‘appendicular’ hypokinetic-rigid signs and with the maximum AP-CG velocity (Table 2; Fig. 3b). The severity of DWML also tended to be related to the FAB score (Table 2; Fig. 3b).

Multiple step-wise regression analysis showed that the severity of falls was dependent upon the N-mesencephalon surface area, whereas the severity of ‘appendicular’ hypokinetic-rigid signs was dependent upon the severity of DWML (Table 2).

## Discussion

In this selected group of elderly HLGD patients, we found a disruption of gait initiation execution with poor postural control, mainly related to bilateral focal atrophy of the MLR. The cognitive deficit and ‘appendicular’ hypokinetic-rigid signs observed in these patients were found to be mainly related to deep periventricular white matter lesions.

HLGD patients were carefully selected using validated criteria [4]. They all displayed a hypokinetic-rigid syndrome with mild frontal-type cognitive disorders [3–5], which are implicated, in part, with the slow gait characteristics of the elderly [34]. These clinical signs suggest that the HLGD patients had suffered a dysfunction of the basal ganglia and/or its output structures, with a striatal dopaminergic denervation, as reported in healthy elderly adults with reduced gait speed [35]. Since neither DAT-Scans nor neuropathological analyses were performed, the possibility of a striatal dopaminergic denervation or a known neurodegenerative parkinsonian syndrome, such as PD, a PSP or MSA, cannot be totally excluded. However, our results suggest that this is unlikely to be the case, because: (1) despite a disease duration > 8 years, no patient developed clinical oculomotor supranuclear palsy, cerebellar deficits or dysautonomia [36]; (2) the gait initiation parameters were different from those reported in neurodegenerative parkinsonian syndromes with no deficit in the preparatory phase [37, 38] and little reduction in postural control [39]; (3) imaging analysis showed no basal ganglia, pons, middle and superior cerebellar peduncles, cerebellum or cortical damage typically seen in such patients with neurodegenerative disorders [30, 40, 41].

### Role of midbrain atrophy in gait and balance disorders of HLGD patients

In our study, gait and balance disorders of HLGD patients, in particular falls and braking deficit, were found to be related to midbrain atrophy. In comparison to controls, HLGD patients showed a significant bilateral reduction in grey matter density of the midbrain area involving both the CN and the PPN. Interestingly, we found a disrupted coupling of preparatory postural adjustments (almost normal) and step execution (severely altered) in our HLGD patients, which could be responsible for falling. In healthy adults, the integrated neural networks for posture and locomotion are activated in parallel, to generate a postural command (for segmental orientation and balance) and a step command [42, 43]. Little is known about the brain regions involved in this integration in humans, however. The MLR would appear to be an ideal candidate to support this physiological function, as its two nuclei, the PPN and the CN, are known to control principally posture and locomotion, respectively [21]. The MLR is also part of a complex network: it receives large inhibitory inputs from the basal ganglia and pontomedullary reticular formation, but also from the spinal cord sensory afferents; it projects in turn excitatory ascending afferents to the basal ganglia and descending projections to several midbrain, pontine and reticular formation nuclei, deep cerebellar nuclei and spinal cord [44]. Lesion or dysfunction of this region could lead to the defective coupling of the postural adjustment-locomotion process found in our patients. Consistent with this idea, patients with focal MLR lesions and parkinsonian patients with a decrease in the grey matter density of the midbrain surface area show similar deficits in the gait initiation process and balance control [11, 12, 45, 46]. In line with these clinical data, experimental lesions of the PPN cholinergic neurons in aged monkeys induce postural and gait deficits [10]. Finally, this suggests that the atrophy of the MLR found in HLGD patients could be the major explanation for the marked alteration of step execution with the loss of coupling between postural adjustment and locomotion.

### Role of deep brain white matter lesions in gait and balance disorders and cognitive deficit of HLGD patients

As previously reported, HLGD patients presented periventricular supratentorial white matter lesions, usually considered to correspond to leukoarariosis [5, 47], that could be related to the cardiovascular risk factors we observed. In our patients, the severity of DWML was significantly related to the ‘appendicular’ hypokinetic-rigid signs and tended to be related to the frontal-lobe–like cognitive deficit (Table 2). This suggests that these symptoms mainly result from a dysfunction and/or disconnection of the loops linking the basal ganglia and the cortical prefrontal areas, as previously reported in elderly subjects with mild hypokinetic-rigid signs [48, 49]. The reduction in gait velocity was also related to the extent of such lesions, as reported in the elderly population [1, 6, 7], suggesting that DWML play a role, at least in part, in the gait velocity reduction observed in these patients [6]. However, the fact that DWML were not found in all patients [5] and that healthy older persons may have DWML without gait or balance disorders [50] indicates that these lesions are not sufficient to provoke HLGD.

### Role of the cortical lesions in gait and balance disorders of HLGD patients

HLGD patients showed a near normal preparatory phase of the gait initiation process (in amplitude and duration), but a severe alteration of gait initiation execution. Nevertheless, patients were able to increase the length and velocity of the first step when asked to, although to a lesser degree than controls. These results suggest that step length and velocity reductions observed in HLGD patients may be related to a decrease in muscle strength, or more specifically, to a defective internal generation of adaptive step length [26, 51]. Nevertheless, a peripheral process could not be totally ruled out, such a strength deficit could be the consequence of the atrophy of the medial primary cortex found in our HLGD patients, as suggested by the presence of an underactivation of the primary motor cortex during walking in PD patients presenting such a deficit [12]. It has also been shown by means of imaging, near-infrared spectroscopic [14, 15, 18, 20] and excitatory transcranial magnetic stimulation [52] techniques that in healthy subjects, this cortical region is involved in gait initiation execution. In addition, HLGD patients have postural control deficits, which may also result, at least in part, from the atrophy seen in the primary motor cortex. Consistent with this hypothesis, TMS activation of the primary motor cortex has been shown to be facilitated by an upright standing position as compared to a supported standing condition [19], but also induced by a signalled postural perturbation [17]. This suggests that the atrophy of the medial primary cortex found in HLGD patients may contribute not only to the reduction of step length [42] and velocity, but also to the balance deficit. Even if the observation of nervous system atrophy does not necessarily reflect the existence of neurodegeneration, as the motor cortex projects directly to the MLR, the atrophy at this level may result from retrograde degeneration [21, 53], leading to a dysfunction of this specific network in HLGD patients. Another explanation for this cortical involvement could be the presence of others neurodegenerative processes, such as β-Amyloid deposition, as reported in healthy older adults [54] and recently shown to be related to postural instability and gait disorders in PD patients [55].

In conclusion, our results indicate that HLGD patients may suffer a neurodegenerative disease that particularly affects the MLR, in combination with DWML. Future longitudinal studies, using new imaging techniques (diffusion tensor imaging, resting state analysis, position emission tomography) and neuropathological examination would be required to further explore the physiopathology of gait and balance disorders of the ageing population.
